# Invited discussant comments during the UCL–Penn Global COVID Study webinar ‘How Do We Trust (Again): Paranoia and Mental Health’: part 2 of 2

**DOI:** 10.14324/111.444/ucloe.100003

**Published:** 2022-11-01

**Authors:** Mitch Cooke

**Affiliations:** 1 Director of Sustainability, Greengage Environmental Ltd., London, UK

**Keywords:** schizotypy, anxiety, depression, stress, loneliness, sleep, Covid-19, mental health, longitudinal health, social value, community consultation, design interventions, green infrastructure, multi-functional benefits, stakeholders

## Abstract

Loneliness has been reported by the UCL–Penn Global COVID Study participants throughout the pandemic year, not surprisingly, although this has been an issue that has been manifesting itself even before the pandemic. In identifying loneliness in communities, the built environment industry and professionals have been looking at how good and targeted design in the public realm and master planning can help to firstly design interventions and secondly orchestrate or manage these spaces in a way that helps create opportunities to address loneliness. Furthermore, how these spaces create opportunities for people to both interact with each other but also interact with the space can help connect people together and with nature/biodiversity. In doing so this also helps to create better health outcomes for mental health and wellbeing, as well as physical health and wellbeing.

Coronavirus (Covid-19) and the associated lockdown periods have caused people to reconnect with local green spaces and has focused the attention to what these spaces provide in terms of opportunities and benefits for people. As a result, the value placed on these and the expectation of how they will provide value to communities is increasing and will continue to increase in the post-Covid-19 world. Better connected, activated and well-structured public realm and green spaces will be central to the development of projects and schemes for housing, and mixed used schemes in the forthcoming years.

**About the study**

The UCL–Penn Global COVID Study^1^ launched in April 2020 is a 12-month longitudinal study of the impact of Covid-19 on social trust, mental health and physical health. In collaboration with six institutions from Italy, Singapore, the United States, China and the United Kingdom,^2^ the study looks at the short- and longer-term effects of Covid-19 on individuals’ mental health and social relationships with others. Survey data were collected at three time-points: 17 April–14 July 2020 (Wave 1), 17 October–31 January 2021 (Wave 2) and 17 April–31 July 2021 (Wave 3).

**About the webinar**

Held online between 2 June and 28 July 2021, the study group presented research data at five online webinars, as part of the UCL Global Engagement Fund sponsorship, to discuss the lessons learned and invited policy makers and other subject experts to speak on the policy relevance and implications of the study findings. The recorded comments from these discussions focusing on the policy relevance and implications of each academic article were recorded as discussant articles and are published in this journal to be read alongside the research article being discussed.

These discussant articles are reviewed by members of the Editorial Board before being published. It is hoped that these discussant articles, read alongside the academic articles, will provide more holistic understanding of the issues at hand, how findings may inform policies in the coming months and/or assist in future crisis management strategies and aid decision-making, in an open and transparent manner.

The study was pre-registered https://osf.io/4nj3g/ on 17 May 2021) and ethical approval was obtained from the University College London Institute of Education Ethics and Review Committee on 8 April 2020 (REC 1331).^1^

**Linked research article**

The linked research article to this discussion article cited here has been published in *UCL Open: Environment* following open peer review and made freely available to read as an open access article. Additionally, all previous versions and peer review reports are freely available to read as open access preprint articles from the journal’s preprint server by following the below DOI link and navigating to the version history of the published research article. Readers can find more information about how peer review works in the journal at ucl.scienceopen.com.

Wong KK, Wang Y, Esposito G, Raine A. A three-timepoint network analysis of Covid-19’s impact on schizotypal traits, paranoia and mental health through loneliness. *UCL Open: Environment*. 2022;(4):17. DOI: https://doi.org/10.14324/111.444/ucloe.000044

**Recorded webinar**

This discussion article comments on the findings presented during the following webinar that has been recorded and made freely available to readers to watch on-demand.

Summer Webinar 2 – Social Trust & Mental Health #GlobalCOVIDStudy. Available from: https://www.youtube.com/watch?v=AcQO1owUNFA

## Introduction

The UCL–Penn Global COVID Study findings, and particularly those on loneliness as an influential variable connecting schizotypy/mistrust to poorer mental health, show the need for interventions that reduce loneliness may improve social trust and mental health. Understanding the profile and make up of communities, and particularly around the instances of loneliness in a community, is a key consideration in the work we do at Greengage, and using design solutions to try to address some of these issues.

Greengage is an independent specialist environmental and sustainability consultancy based in London and working nationwide. We are a sustainability consultancy that works in partnership with our clients to deliver truly sustainable solutions, and our advice is based on delivering net gains in environmental, social and economic terms. We work collectively across design and client teams to ensure that the advice we provide assists our clients in delivering resilient places that benefit both people and nature.

We believe in delivering work that makes a difference to people’s lives. We are sustainably led in that we believe that sustainability and the social impact of the development is what leads the design and the impact assessments and is what creates healthy, resilient places for both people and nature.

By looking at the socio-economic profiles of communities in areas where development is planned, an understanding of the challenges and issues within the community can be mapped and understood. This, in conjunction with extensive public engagement and consultation, can identify the needs of the community and what the development can and should deliver in social value benefits. In urban – and in London particularly – communities can be dislocated and certain sections of communities can be divided from networks that would normally enable social interaction and generational mixing. The prevalence of loneliness in urban settings is high where social fabric is low and the integration of design-led solutions can help improve factors that address loneliness in communities, as well as help provide interventions that benefit active travel, connecting with nature, that also have positive health outcomes, improving mobility and mental health factors. These interventions vary in scale and nature and are focused on the spaces between the buildings (although there are a range of factors associated with homes, schools and places of work and worship that are understood to promote healthy buildings)^3^. As such, our role as practitioners is as facilitators in ‘good’ design in the right locations, aimed at delivering set objectives around inter-generational mixing, social cohesion, active travel and addressing mental health. It should be recognised that whilst these interventions are physical features, the orchestration of place also has a role in ensuring that the design interventions are ‘active’ and not passive in their use. We facilitate input therefore from architects, master planners, urban designers as well as landscape practitioners, ecologists, community engagement specialists as well as members of the community and other stakeholders. These interventions can be hard – seating, play equipment, meanwhile use, sports facilities – or soft such as urban greening, local food production, allotments, grassed and ‘wild’ spaces designed for biodiversity^4,5^. The activation or orchestration of spaces can be events, activities, gatherings and wayfaring/natural indication that the ‘activity’ is permitted and encouraged. An acknowledgement of ownership over a space or activity should be inherent to its design and operation.

## Discussant comments – Q&A

**Q:** A question was asked about the move towards more mixed communities where proximity of work to homes, schools and retail offerings and if this is an upward trend.

**A:** Indeed we are seeing a drive particularly around high streets and the High Street Taskforce that was created in early 2020 aimed to help address the issue of high streets losing local traders, individuality and becoming centres for anti-social behaviour. Covid-19 has focused the attention on this further with plans for town centres now looking at how a range of uses including residential and offices can return to the high street. It is acknowledged that online retail will continue to play a big part in future shopping habits but that shoppers also want a ‘retail’ and high street experience, and so active high streets will need to help deliver this with a range of mixes to include cultural and entertainment, as well as dining and hospitality. Residential use in town centres will also return and high streets will need to integrate aspects into their design for residents to mix, meet and dwell to create activation in these spaces that will draw in ‘shoppers’ for their high street experience.

**Q**: A further question was asked about whether our industry has increased through the pandemic.

**A:** The short answer to this is yes, in that it has made people value the ‘spaces’ and in particular well designed, multifunctional greenspace that integrates biodiversity and connects with nature. There has and continues to be a great deal of focus on the impacts of lockdown on communities’ collective and individuals’ mental health as a result of isolation and disconnection from their social networks. We all saw an increase in the use of these greenspaces – even small and often overlooked greenspaces – and the value that people placed on them as places of refuge. Increasingly people are now demanding that these spaces are multifunctional and created to provide a range of benefits rather than accepting poorly designed spaces.

**Q**: Will there be greater opportunity to work with a range of disciplines in this work in the future?

**A:** Yes – there is an increasing need to rely upon an evidence-based and reliable mechanism so that developer contributions paid through the planning process can be shown to deliver the benefits that are set in in the design process^6^. This will necessitate the input from a range of professional interests and backgrounds, not only in the design of these features and interactions, but in monitoring of the outcomes from these interventions and the ‘value’ these deliver for communities and people.

## Summary

Community mapping and socio-economic profiling of communities and stakeholder engagement around and affected by proposed developments can indicate what design interventions are necessary to define social value benefits and outcomes. There are a range of design guides from the Centre for Active Design, Sports England’s Active Design Principles, Transport for London (TfL) Healthy Streets that set out what a healthy community should and could integrate. These include features such as connectivity, community facilities, good air quality and noise environments, greenspaces, that have a range of mixed uses, arts and culture and accessible spaces. Access to local meaningful employment, and feeling safe and secure are also key in designing for a healthy community. The opportunity for projects to deliver these is often set out when projects are conceived but they are not often based on the local needs of communities or individuals. The mapping and engagement process enables these to be aligned and for set objectives to be agreed, and for mechanisms for their delivery and effectiveness set out and funding committed to. In that way practitioners can deliver sustainable communities that not only have community resilience but have wider societal benefits through reduced demand for social and health care.

## Notes

1 Wong KK, Raine A. Covid-19: Global study of social trust and mental health. Available from: https://globalcovidstudy.com [Accessed: 11 November 2021].

2 Wong KK, Raine A. Covid19: Global social trust and mental health study. 2020. https://doi.org/10.17605/OSF.IO/FE8Q7.

3 The Elephant & Castle Partnership. Available from: https://www.elephantandcastle.org.uk/about-elephant-and-castle/.

4 The Old Kent Road. Available from: https://www.southwark.gov.uk/planning-and-building-control/planning-policy-and-transport-policy/development-plan/area-action-plans-section/old-kent-road-aap.

5 Clapham Park. Available from: https://yourclaphampark.co.uk/.

6 Silvertown. Available from: https://www.silvertown.co.uk/.

